# Early childhood height is a determinant of young adult stature in rural Nepal

**DOI:** 10.1186/s12889-024-19469-8

**Published:** 2024-07-30

**Authors:** Jiaxin Chen, Ramesh K. Adhikari, Lee S-F Wu, Subarna K. Khatry, Parul Christian, Steven C. LeClerq, Joanne Katz, Keith P. West Jr.

**Affiliations:** 1 Center for Human Nutrition, Department of International Health, Johns Hopkins Bloomberg School of Public Health, Baltimore, MD USA; 2 Institute of Medicine, Tribhuvan University, Kathmandu, Nepal; 3 Nepal Nutrition Intervention Project – Sarlahi (NNIPS), Kathmandu, Nepal; 4 Global Disease Epidemiology and Control Program, Department of International Health, Johns Hopkins Bloomberg School of Public Health, Baltimore, MD USA

**Keywords:** Linear growth, Child height, Adult height, Nepal

## Abstract

**Background:**

Does preschool height predict adult stature in undernourished settings? The extent to which preschool length or height forecasts young adult stature is unclear in chronically undernourished populations.

**Methods:**

In 2006-8, we assessed height in a cohort of 2074 young adults, aged 16–23 years, in rural Nepal who, as preschoolers (*≤* 4 year), were measured at baseline and again 16 months later during a vitamin A supplementation trial in 1989-91. We assessed by linear regression the ability of preschool length (L, measured < 24 mo) or height (Ht, 24–59 mo), at each year of age to predict 16–23 year old height, adjusted for month of young adult age, interval duration (in months), caste, preschool weight-for-height z-score and, in young women, time since menarche, marriage status and pregnancy history.

**Results:**

Young women were a mean of 0.81, 1.11, 0.82, 0.24, 0.44 cm taller (all *p* < 0.01) and young men, 0.84, 1.18, 0.74, 0.64 and 0.48 cm taller (all *p* < 0.001) per cm of attained L/Ht at each successive preschool year of age and, overall, were 2.04 and 2.40 cm taller for each unit increase in preschool L/Ht z-score (L/HAZ) (both *p* < 0.001). Coefficients were generally larger for 16-month follow-up measurements. The percent of young adult height attained by children with normal L/HAZ (>-1) increased from 38–40% mid-infancy to ∼ 69–74% by 6 years of age. By 3–6 years of age heights of stunted children (L/HAZ<-2) were consistently ∼ 4–7% lower in their young adult height versus normal statured children. There was no effect of preschool vitamin A receipt.

**Conclusions:**

Shorter young children become shorter adults but predictive effects can vary by sex, age assessed, and may be influenced by year or season of measurement.

**Supplementary Information:**

The online version contains supplementary material available at 10.1186/s12889-024-19469-8.

## Background

Adult height represents accumulated linear growth from fetal life through infancy, childhood and adolescence [1–3]. Although velocities uniformly decrease with age until pubescence, within supportive environs of routine dietary adequacy and generally good health, children are expected to reach around 70% of their final height by age 7 and about 80% by age 10 years [4]. Approximately 15% of adult height is attained during adolescence, most of which occurs during puberty (ages 10 to ∼ 14 years) [5–8]. For example, in British and Denmark cohort studies, adult height was strongly predicted from child height at age 7 for both sexes, [1, 4] emphasizing the importance of adequate linear growth in childhood toward establishing final adult height.

In less supportive environs, where early childhood nutritional status is often characterized in terms of wasting and stunting, it is of interest to quantify, if possible, the extent to which attained preschool growth anticipates adult stature. In an early 21st century longitudinal study in India, the association between preschool height and adult height strengthened with increasing age, reaching a correlation coefficient of 0.7 by age five and continued increasing after puberty [9]. Expressing achieved linear growth as length for age z-scores (LAZ), the Cohorts Study, which has followed growth of 4659 children from infancy into adulthood in Brazil, Guatemala, India, the Philippines, and South Africa, has reported a mean increment of 0.45 height-for-age z-score (HAZ) at ages 15 to 32 years per z-score increment at 12 months of age [10]. In Pelotas, Brazil Cohorts Study investigators reported that heights of young women were, on average, 0.8 cm taller at 19 years per cm of attained height at four years of age [11]. Panels of *synthetic* cohorts have been created in 21 low-middle income countries (LMICs) by matching distributions of preschool child and adult height aligned by chronological years of birth from repeated Demographic and Health Surveys (DHS) conducted over a period of three decades. Based on these aligned cross-sectional datasets, Karra and Fink have estimated that each unit increase in HAZ under 5 years of age is associated with an estimated increase in adult height of 2.92 cm and 1.88 cm in men and women, respectively [12].

In this paper, we contribute predictive relationships between preschool length (< 24 months) and height (24–59 months) and late adolescent/young adult^1^ height in a population-based cohort of individuals who, as preschoolers, participated in a vitamin A supplementation trial from 1989 to 1991. Study participants lived in the rural District of Sarlahi, lying along the southern plains (*Terai*) of Nepal [13], a setting where moderate undernutrition remains a chronic public health concern [14]. We report estimates adjusted only for study design factors and, in addition, adjusted for nutritional and socioeconomic status and, for young women, age at menarche, marital status and pregnancy history (that may affect adolescent growth), and to a limited extent season and year of measurement. The influence of cluster-randomized supplement allocation during the original placebo-controlled vitamin A trial was also examined.

## Methods

This secondary data analysis was based on longitudinal records of a subset 2074 children, within a growth sub-study cohort of 4765 children *≤* 60 months of age, who were enrolled from September to December 1989 into a large cluster-randomized, placebo-controlled vitamin A supplementation trial (total *n* = 28,630) that involved home visits with supplement provision every 4 months [13]. The trial was stopped following completion of a 16-month assessment (in January-April 1991) due to an observed 30% reduction in child mortality. The entire trial cohort was revisited to receive a vital, health and nutritional assessment ∼ 16 years later. This analysis was restricted to children who were assessed at either the trial’s baseline or 16-month visit, and had complete data at young adult follow-up.

### Original trial

The design of the original trial and its findings on the efficacy of vitamin A supplementation in reducing child mortality and xerophthalmia and improving growth have been previously reported [13, 15–18]. Among 261 subdistrict wards (clusters) that were mapped, addressed and randomized for the trial, 40 were randomly sampled, stratified by allocation and geographic area, for all resident, enrolled children at baseline to receive an ocular exam and be measured for anthropometry. At both baseline (Sep – Dec 1989, pre-harvest) and 16-month visits (Jan – Apr 1991, post-harvest), children were weighed either naked or lightly clothed on hanging Salter spring scales (West Bromwich, England), with values recorded to the nearest 0.1 kg after the pointer was stable for ≥ 2 s. Recumbent length (L, for children aged < 24 months) and standing height (Ht, for children *≥* 24 months) were measured and recorded independently 3 times to the nearest 0.1 cm on wooden boards with footplate, sliding head block and attached steel tape to the nearest 0.1 cm (Weigh and Measure LLC, Olney, MD, USA), with the median taken as actual. Height-for-age (HAZ) or length-for-age (LAZ), weight-for-height (WHZ) and weight-for-age (WAZ) z-scores were derived from the WHO reference population [19]. Mid-upper arm circumference and tricipital and subscapular skinfold measurements and 1-week morbidity histories were also measured at each visit. Household socioeconomic characteristics, including caste membership (Brahmin, Chhetri, Vaishya, Shudra, and non-Hindu), were assessed shortly after the baseline visit. The trial was approved by the Nepal Medical Research Council (Kathmandu, Nepal) and the Johns Hopkins Joint Committee on Clinical Investigation (Baltimore, MD, USA).

### Follow-up study

Households of children who completed the 1989-91 trial were initially revisited by field teams in 2006-8 to record vital status, confirm identity and residency, obtain informed consent for a follow-up assessment, and be scheduled for a home-based assessment. Children in households that had permanently moved from the district were followed for vital status, if possible, but excluded from assessment protocols.

Cohort follow-up procedures and findings related to multiple health outcomes have been reported [20–23]. Briefly, data were collected on household socioeconomic variables, adolescent/young adult participant health and marital status, and for females, recalled age (in years) at menarche, marriage status and if married, pregnancy history. Anthropometry was conducted by trained, original trial field staff who measured participant height with shoes removed on a locally constructed stadiometer, recorded the median of 3 measurements to the nearest 0.1 cm, and weight without shoes and light clothing recorded from a Seca digital scale (Seca, Chino, CA, USA) to the nearest 0.1 kg. Body mass index (BMI) was calculated as weight in kg/height in m². Caste was assumed to be unchanged from the original trial. As month and year of birth had been recorded with assistance of event calendars during the trial, ages were considered accurately known, ranging from 16 to 23 years, converted to age in months which was used to further refine age-adjusted size measures. All follow-up assessments by staff were conducted blinded to supplement allocation, and preschool nutritional, health and household socioeconomic status data from the original trial. The follow-up study was approved by the Nepal Health Research Council (Kathmandu, Nepal) and the Institutional Review Board at Johns Hopkins Bloomberg School of Public Health (Baltimore, MD, USA).

### Statistical analysis

Anthropometric data at baseline (Sept-Dec 1989), derived from preschoolers in a highly compliant, random selection of wards throughout the rural study area of ∼ 250 sq km, when paired to 2006-8 follow-up data, was considered the primary dataset for assessing preschool child growth: follow-up statural associations. Sixteen-month follow-up data collected from Jan-April 1991 in the same cohort of children provided further opportunity to examine the same relationships involving a different season and year of initial assessment. Age-sex specific anthropometric z-scores for length-for-age (LAZ), height-for-age (HAZ), weight-for-height (WHZ) and weight-for-age (WAZ) were derived from the WHO Multicenter Growth Reference Study [19].

Sex-specific, linear regression models for each preschool year of age were employed to quantify the associated effect size of preschool length or height on young adult height. As the 2006-8 follow-up study was conducted over a period of ∼ 1.5 years, without attempting to match visits to month or sequence of preschool assessment, all models are adjusted for late adolescent/young adult age in months to account for this source of variation in age.

For the baseline-derived analysis, children at exactly 60 months of age (*n* = 26) were dropped to remain consistent with ages being < 5 years. An initial linear regression (Model 1) was introduced to adjust for late adolescent/young adult age and interval duration between preschool assessment and the 2006-8 follow-up. Multivariable regression analysis (Model 2) further included preschool WHZ and caste as covariates, and for females, marriage status, pregnancy history and time since menarche. Preschool WHZ has been included to adjust coefficients for initial wasting status and as a proxy for other associated anthropometric measures (i.e. mid-upper arm circumference and skinfolds). Caste, acting as a strong indicator of socio-economic status, has been added as a covariate to broadly adjust for social, economic, local environmental risk factors [24–27]. As age at marriage and pregnancy may be associated with sexual maturation, age at menarche [28], growth and development [29, 30], and as pregnancy can have a suppressing effect on late adolescent growth [31], these factors have also been included in the models. As history of menarche was obtained by year of age, and as age of menarche follows a sigmoidal curve [32], we assigned mid-year ages for girls who reported menarche at 14 (i.e., 174 mo) and 13 (162 mo) years, progressively shifting the assigned age to older months for girls reporting menarche at 12, 11 and 10 years, and toward younger months for girls reporting menarche at ages 15, 16 and older years. To aggregate effect sizes across all preschool ages, we repeated analyses replacing preschool L or Ht measurements in cm with L/HAZ as the independent variable, accounting for age.

Identical growth interval analyses were carried out using measurements taken at the 16-month trial visit, which encompassed the winter post-harvest season of 1991, in contrast to the initial assessments taken during the post-monsoon, preharvest season of 1989. Using this interval also excluded infancy with aging of the cohort and generated estimates for each 1-year stratum from 1 to 6 years of age. Children with complete data at 16-month visit and follow-up during late adolescence to young adulthood were included in this second evaluation. Since 16-month follow-up is closer by this amount of time to the young adult measurement, an interval duration covariate was further introduced to both Model 1 and Model 2 to account for this potential source of variation due to design. The potential influence of vitamin A vs. placebo supplementation during the original trial was examined by including ward allocation as a covariate in preschool-to-late adolescent/young adult height models.

We further estimate the percentage of young adult height attained at each years of age at the baseline and 16-month visit, stratified by sex and L/HAZ [i.e., > -1 z (normal length or height), -2 to -1 z (mildly stunted) and < -2 z (moderately to severely stunted)] to maximize use of available age-specific data and enable comparison with data from better nourished societies.

Complete data during the original trial baseline or 16-month visit, and at the young adult follow-up were required for inclusion in this analysis. Given this requirement, we examined the potential for bias by comparing preschool household socioeconomic variables assessed during the original trial between participants included in this analysis and those excluded due to incomplete data or reportedly being alive but unreachable at follow-up. Significance of distributional differences was compared by the Chi-squared test. Household characteristics of children who had died between the end of trial and time of young adult follow-up are also presented.

All analyses were conducted using Stata, version 17.0, software.

## Results

**Fig. 1:**
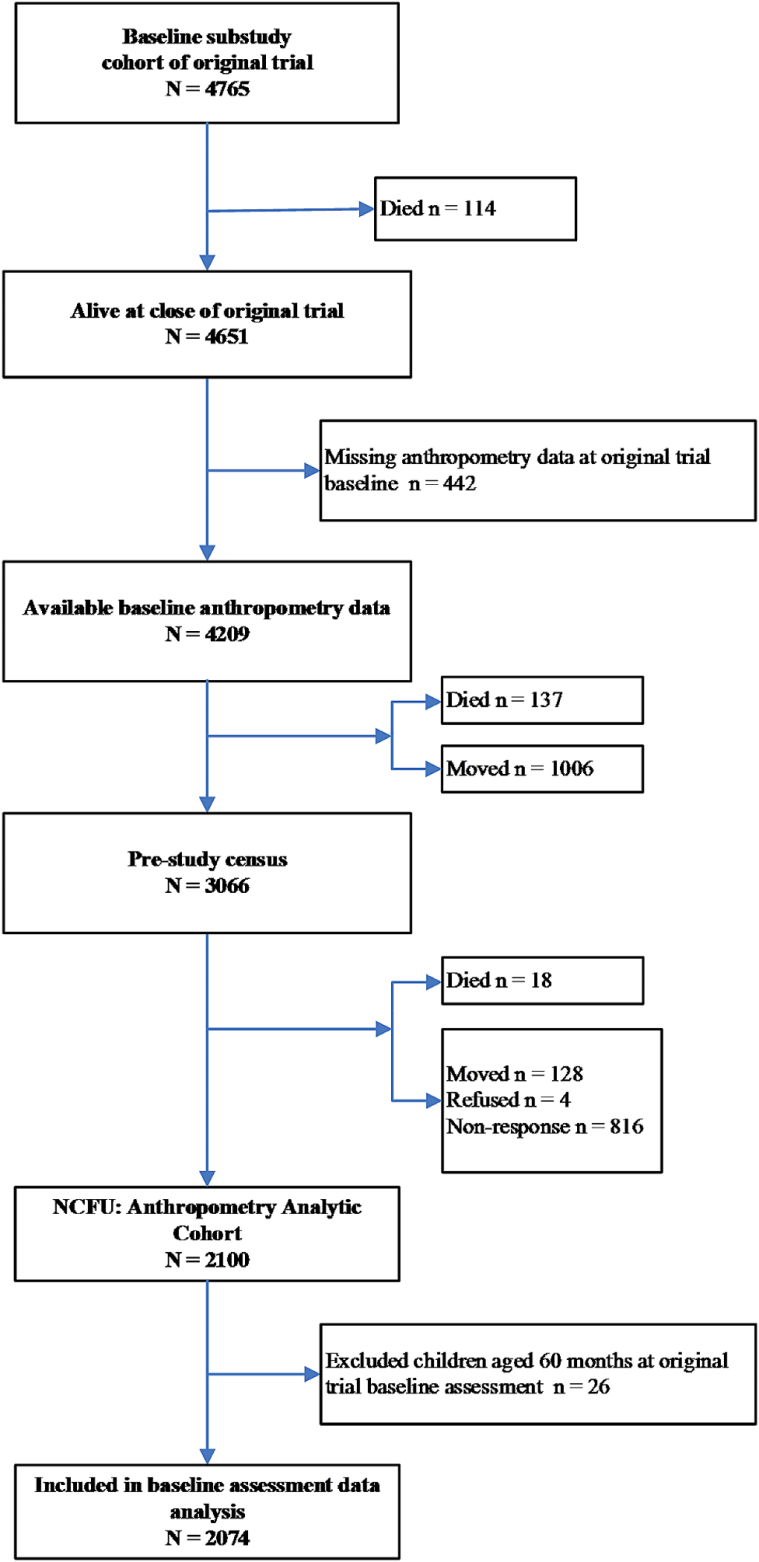
Flowchart of Participants as Preschoolers (< 60 months) at Baseline (in 1989), and as Censused (in 2006) and Followed-up as Late Adolescents and Young Adults (in 2006–2008), Nepal Nutrition Intervention Project-Sarlahi, Nepal

Initially, 4765 children aged 0–60 months were included into this analytical substudy, of whom 4651 were alive at the end of the original trial, among whom 442 were missing anthropometry at baseline and excluded. By the time of either the census or follow-up assessment ∼ 16 years later, an additional 155 children had been identified as deceased and 1138 had moved or refused and were considered lost. At follow-up, 816 were reported to be resident but not measured after *≥* 2 repeat visits, leaving 2100, or 50% of 4214 surviving and measured children who exited the original trial (Fig. 1). Sex was available for 858 girls and 1216 boys, representing the number of participants in this analysis. With respect to assessed household socio-economic characteristics at the time of the original survey, surviving individuals included and excluded from the analytical cohort were comparable (Table 1), providing no evidence of selection bias based on factors in early childhood.

**Table 1:** Baseline Comparison of Social and Economic Characteristics of Rural Nepalese Participants and Non-participants ^1^ in the Analytical Cohort from the Original Vitamin A Supplementation Trial, September-December, 1989

	Analytical cohort	Non-participants^1^	Deceased^2^
**Number**	2074 (100%)	2422 (100%)	269 (100%)
Household members*			
0–5	655 (32%)	858 (35%)	108 (40%)
6–10	1163 (56%)	1224 (51%)	136 (51%)
more than 10	253 (12%)	307 (13%)	24 (9%)
Don’t know	3 (0%)	33 (1%)	1 (0%)
Caste*			
Brahmin	271 (13%)	244 (10%)	16 (6%)
Chhetri	203 (10%)	176 (7%)	9 (3%)
Vaiysha	1341 (65%)	1553 (64%)	167 (62%)
Shudra	157 (8%)	254 (11%)	40 (15%)
Non-Hindu	99 (5%)	163 (7%)	36 (13%)
Don’t know	3 (0%)	32 (1%)	1 (0%)
**House Characteristics**			
Ground floor*			
No walls	30 (1%)	29 (1%)	1 (0%)
Thatch, grass, sticks, branches	1073 (52%)	1396 (58%)	179 (67%)
Katcha	488 (24%)	481 (20%)	45 (17%)
Wood planks	399 (19%)	374 (15%)	39 (15%)
Pakka	81 (4%)	110 (5%)	4 (1%)
Don’t know	3 (0%)	32 (1%)	1 (0%)
First floor*			
No walls	1255 (61%)	1651 (68%)	215 (80%)
Thatch, grass, sticks, branches	153 (7%)	163 (7%)	7 (3%)
Katcha	229 (11%)	222 (9%)	16 (6%)
Wood planks	411 (20%)	324 (13%)	29 (11%)
Pakka	23 (1%)	30 (1%)	1 (0%)
Don’t know	3 (0%)	32 (1%)	1 (0%)
**Roof** *			
No roof or plastic	4 (0%)	3 (0%)	0
Thatch or grass	807 (39%)	1176 (49%)	152 (57%)
Tile	1217 (59%)	1169 (48%)	113 (42%)
Tin	5 (0%)	9 (0%)	1 (0%)
Pakka	38 (2%)	33 (1%)	2 (1%)
Don’t know	3 (0%)	32 (1%)	1 (0%)
**Rooms**			
0–4	1966 (95%)	2270 (94%)	257 (96%)
5 or more	105 (5%)	120 (5%)	11 (4%)
Don’t know	3 (0%)	32 (1%)	1 (0%)
Water*			
Tube well or tap	1028 (50%)	1235 (51%)	150 (56%)
Ring well	919 (44%)	933 (39%)	100 (37%)
Other	124 (6%)	222 (9%)	18 (7%)
Don’t know	3 (0%)	32 (1%)	1 (0%)
**Latrine**			
No latrine	1957 (94%)	2256 (93%)	264 (98%)
Pit latrine	87 (4%)	89 (4%)	2 (1%)
Water-sealed toilet	27 (1%)	45 (2%)	2 (1%)
Don’t know	3 (0%)	32 (1%)	1 (0%)
**Household Ownership**			
Cattle*			
None	538 (26%)	819 (34%)	107 (40%)
1–9	1418 (68%)	1472 (61%)	157 (58%)
10 or more	115 (6%)	99 (4%)	4 (1%)
Don’t know	3 (0%)	32 (1%)	1 (0%)
Goats*			
None	971 (47%)	1251 (52%)	147 (55%)
1–5	910 (44%)	922 (38%)	104 (39%)
6 or more	190 (9%)	217 (9%)	17 (6%)
Don’t know	3 (0%)	32 (1%)	1 (0%)
Bullock carts*			
None	1693 (82%)	2049 (85%)	239 (89%)
Any	378 (18%)	341 (14%)	29 (11%)
Don’t know	3 (0%)	32 (1%)	1 (0%)
Bicycles*			
None	1628 (79%)	1955 (81%)	239 (89%)
Any	443 (21%)	435 (18%)	29 (11%)
Don’t know	3 (0%)	32 (1%)	1 (0%)
**Radios**			
None	1578 (76%)	1838 (76%)	232 (86%)
Any	493 (24%)	552 (23%)	36 (14%)
Don’t know	3 (0%)	32 (1%)	1 (0%)
**Watches**			
None	1524 (74%)	1798 (74%)	218 (81%)
Any	547 (27%)	592 (24%)	50 (19%)
Don’t know	3 (0%)	32 (1%)	1 (0%)
**Domestic Servants**			
None	1858 (90%)	2161 (89%)	254 (94%)
Any	213 (10%)	229 (9%)	14 (5%)
Don’t know	3 (0%)	32 (1%)	1 (0%)

^1^Non-participants were excluded due to incomplete data or lost to measurement follow-up prior to late adolescent/young adult assessment

^2^Individuals who died between the baseline assessment of the original trial and prior to late adolescent/young adult follow-up (see Fig. 1)

*p < 0.05 by χ ^2^ (with 1 to 4 df) testing differences between participants and non-participants in the analytical cohort

Testing excluded “Don’t know”. For “Roof” testing, “No roof or plastic” combined with “Thatch or grass”

Table 2 summarizes preschool and young adult growth characteristics of the cohort. At baseline preschool heights increased with age, with boys being a mean of 1.1 to 2.8 cm taller than girls in each age group. Among young adult females stratified by their preschool ages during the trial, mean heights are consistently at ∼ 151 cm, while males within the same original strata were, on average, 11–12 cm taller.

**Table 2:** Preschool Length or Height^1^ (L/Ht, cm) and its Association with Height (cm) at 16–23 Years of Age by Age and Sex in a Population Cohort in the Terai of Nepal

Preschool age (yr)	*N*		Baseline height Mean (SD)		Young adult height Mean (SD)		Young adult age in month Median (IQR)		Young adult height/preschool L/Ht [b (SE)]			
	Female	Male	Female	Male	Female	Male	Female	Male	Female		Male	
									Model 1^2^	Model 2^3^	Model 1^2^	Model 2^3^
< 1	189	237	60.3 (5.9)	62.0 (6.6)	151.3 (5.7)	161.9 (6.3)	215 (210–219)	217 (211–221)	0.86 (0.14) *	0.81 (0.16) *	0.84 (0.11) *	0.84 (0.12) *
1	211	235	72.3 (4.7)	74.1 (4.3)	150.9 (6.3)	163.1 (6.3)	227 (223–232)	229 (223–233)	1.09 (0.09) *	1.11 (0.10) *	1.19 (0.09) *	1.18 (0.09) *
2	154	256	80.2 (4.4)	81.3 (4.4)	152.0 (6.0)	163.5 (6.2)	238 (234–243)	240 (235–245)	0.84 (0.09) *	0.82 (0.10) *	0.75 (0.09) *	0.74 (0.09) *
3	173	246	86.6 (6.0)	89.4 (5.8)	150.7 (6.1)	163.3 (6.9)	252 (247–256)	254 (249–259)	0.33 (0.08) *	0.24 (0.08) †	0.68 (0.07) *	0.64 (0.07) *
4	131	242	94.1 (5.6)	95.8 (5.2)	151.5 (5.7)	164.2 (6.4)	263 (260–268)	266 (261–270)	0.29 (0.09) †	0.44 (0.09) *	0.50 (0.08) *	0.48 (0.07) *

^1^Recumbent length (L) for children aged < 24 months. Standing height (Ht) for children > 24 months

^2^Coefficient adjusted for interval duration in months and older adolescent/young adult age in months

^3^Coefficient further adjusted for caste, preschool weight-for-height (WHZ) z-score, and for female, also included time since menarche in month, and marriage and pregnancy status (see supplemental Table 1 for distributions)

*p < 0.001, †*p* < 0.01, ‡*p* < 0.05

Young adult height was positively associated with preschool length/height. In linear regression, Model 1, which adjusts for the preschool-to-adult time interval and young adult age, the beta-coefficient (b) revealed a height at 16 to 23 years that was 0.86 –0.84 cm and 1.09–1.19 cm higher per cm of attained length during infancy and 2^nd^ year of life, respectively, and a b = 0.84 and 0.75 cm at age 2 yrs, for females and males, respectively. A strong association generally remained at 3 and 4 years of age for boys (Δ = 0.68 and 0.50 cm), but was weaker for girls (Δ = 0.33 and 0.29 cm, respectively). In Model 2 (Table 2), increments were further adjusted for potential effects of several variables summarized in Supplemental Table 1 (See Supplemental Table 1, Additional File 1), including preschool WHZ and household caste, for young women, marital status, time since menarche and pregnancy history. None of these adjustments, however, notably changed slope coefficients of young adult height on preschool length or height from Model 1 for either sex, suggesting a lack of confounding among model covariates.

Applying the same analyses with data from 16-month visits instead of baseline, 1999 children (See Supplemental Fig. 1, Additional File 1) were stratified by the cohort’s 16-month end-of-trial ages of 1, 2, 3, 4, 5, and 6 years. Qualitatively similar but stronger associations were observed, reflected by Model 2 regression beta-coefficients of 1.06, 1.00, 1.16, 0.49, 0.50, and 0.46 cm/cm among young women (all *p* < 0.001), and 1.06, 1.09, 0.89, 0.74, 0.66, and 0.61 cm/cm among young men (all *p* < 0.001), respectively (See Supplemental Table 2, Additional File 1). None of the above same covariates added into Model 2 notably changed the results.

Expressing attained preschool L/Ht as height-for-age z-scores (L/HAZ), Table 3 allowed a sex specific effect size to be estimated for all ages combined, adjusted for the same covariates in Models 1 and 2. For each unit increase in LAZ or HAZ, females and males were an additional 2.04 and 2.40 cm taller at 16–23 years of age. The effect size was stronger for the 16-month follow-up than baseline assessment, revealing young adult height increments of 2.95 and 3.39 cm per L/HAZ score for females and males, respectively (See Supplemental Table 3, Additional File 1). Modeled young adult height distributions by caste, marriage, and pregnancy factors are summarized in Supplemental Table 4.

**Table 3:** Preschool L/HAZ and its Association with Height (cm) at 16–23 Years of Age in a Population Cohort in the Terai of Nepal

Model Covariates	Girl				Boy			
	Model 1^1^		Model 2^2^		Model 1^1^		Model 2^2^	
	*N* = 856		*N* = 798		*N* = 1215		*N* = 1206	
	b (SE)	*P*-value	b (SE)	*P*-value	b (SE)	*P*-value	b (SE)	*P*-value
**Preschool L/HAZ**	2.04 (0.14)	< 0.001	2.04 (0.15)	< 0.001	2.46 (0.13)	< 0.001	2.40 (0.13)	< 0.001
**Young adult age**,** mo**	0.05 (0.01)	< 0.001	0.08 (0.02)	< 0.001	0.07 (0.01)	< 0.001	0.07 (0.01)	< 0.001
**Interval duration**,** mo**^3^	-0.02 (0.04)	0.64	-0.01 (0.04)	0.87	-0.05 (0.03)	0.12	-0.05 (0.03)	0.11
**Marriage/pregnancy status** ^4^								
Married but not pregnant	-	-	0.80 (0.74)	0.28	-	-	-	-
Married and pregnant	-	-	-0.33 (0.47)	0.48	-	-	-	-
**Time since menarche**,** mo**^5^	-	-	-0.04 (0.01)	0.001	-	-	-	-
**Preschool WHZ**	-	-	-0.42 (0.22)	0.05	-	-	-0.64 (0.19)	0.001
**Caste** ^6^								
Chhetri	-	-	-0.82 (0.73)	0.27	-	-	-0.68 (0.69)	0.33
Vaiysha	-	-	-2.54 (0.55)	< 0.001	-	-	-2.59 (0.50)	< 0.001
Shudra	-	-	-5.45 (0.85)	< 0.001	-	-	-5.41 (0.74)	< 0.001
Non-Hindu	-	-	-4.45 (1.05)	< 0.001	-	-	-3.34 (0.84)	< 0.001
**cons_**	148.8 (7.6)	< 0.001	142.03 (7.9)	< 0.001	162.8 (6.6)	< 0.001	164.57 (6.5)	< 0.001

^1^Coefficient adjusted for interval duration in months and older adolescent/young adult age in months

^2^Coefficient further adjusted for caste, preschool weight-for-height (WHZ) z-score, and for female, also included time since menarche in month, and marriage and pregnancy status (see supplemental Table 1 for distributions)

^3^Time interval between baseline and young adult assessment

^4^“Neither married nor pregnant” was the comparison group. Adjusted mean young adult heights by marriage and pregnancy status were listed in Supplemental Table 5

^5^Girls who haven’t menarche yet (*N* = 7) were excluded in Model 2

^6^“Brahmin” was the comparison group. Adjusted mean young adult heights by caste were listed in Supplemental Table 5

**Fig. 2:**
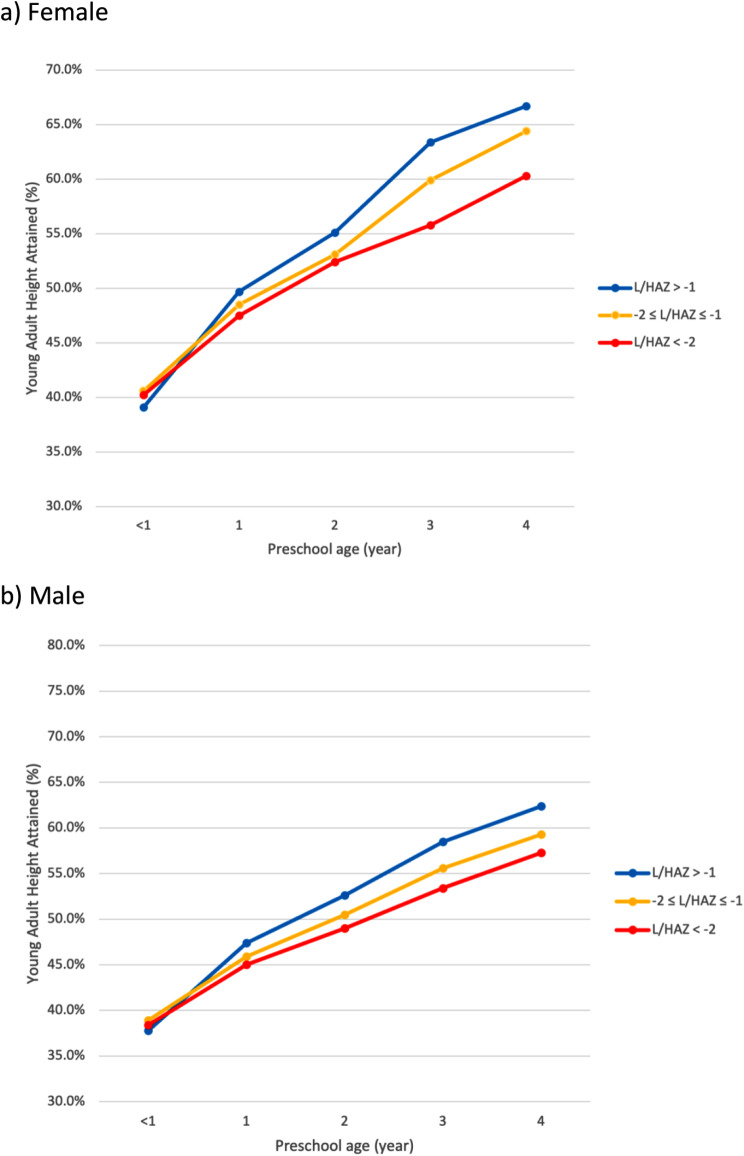
Percent of Height at 16–23 Years of Age Attained by Year of Early Childhood Age at Baseline, by Sex and Preschool Length/Height for Age Z-score in a Population Cohort in the Terai of Nepal

Figure 2 and Supplemental Fig. 2 present the percentage of height at 16–23 years of age that was attained at each early childhood year of age, stratified by L/HAZ and sex. Children (female|male) of normal height (L/HAZ>-1) achieved 39|38, 50|47, 55|53, 63|59, and 67|62 percents of their young adult height at each year of age from infancy (0) through 4 yrs of age (See Supplemental Table 5, Additional File 1). Data from 16-month visit complement height attained by children aged 5 and 6 yrs, at which girls with normal height gained 72% and 74%, and boys, 66% and 69% of their young adult height, respectively. For both sexes by ages 3–6 years, children in the mid-range of L/HAZ (-1 to -2 Z) had attained ∼ 1–3% less, and stunted children (L/HAZ <-2) ∼ 4–7% less of their young adult height than children of normal height.

There was no cohort effect due to vitamin A versus placebo supplement receipt during the original trial.

## Discussion

In this chronically undernourished rural South Asian setting, height attained by late adolescence through young adulthood was positively associated with preschool length below two years or height at older ages, with regression coefficients derived from baseline measurements of the original trial ranging from 0.24 to 1.11 and 0.48 to 1.18 cm increases in young adult height per preschool cm in girls and boys, respectively. Effect sizes, determined from measurements taken 16 months later in the same cohort but in a different season (winter, post-harvest vs. post-monsoon, pre-harvest) of the following year, were comparable, ranging from 0.46 to 1.16 and 0.61 to 1.09 cm in young adult height per cm, respectfully. Linear growth appeared to most efficiently translate to young adulthood height in the 1st three years of life, during which most coefficients exceeded 1 cm of young adult height per cm of length or height. Coefficients derived from ages four to six years were generally lower, 0.44 to 0.50 and 0.48 to 0.74 cm per cm in girls and boys, respectively (Table 2 & Supplemental Table 2).

We also examined associations of early childhood length or height for age z-scores for each sex, allowing all early child ages to be combined, finding late adolescent and young adult stature to increase by 2.04 and 2.40 cm per standardized normal deviate in length or height for age in girls and boys, respectively. Caste, as a strong proxy for socioeconomic status [24–27], preschool weight for height z-score, time since menarche, after which growth slows in girls [28], marriage status, the age at which can be influenced by maturation and growth of girls [29, 30], and history of pregnancy which may slow maternal growth [31], exerted no influence on this relationship.

Our longitudinal cohort findings support those from a synthetic panel cohort study derived from 21 cross-sectional Demographic Health Surveys in low- and middle-income countries [12]. This study found that among children under five years, there was an estimated increase of 2.01 cm (2.92 cm and 1.88 cm for male and female, respectively) in adult height for each additional z-score increase in preschool height-for-age. Given that the average adult male and female height was approximately 164 cm and 151 cm in our study, the increments per HAZ represented 1.8% and 1.2% of final adult height. The synthetic cohorts for each country were assumed to have maintained residence in their same locales without loss to follow-up. Similarly, our study tracked the same children over time, excluding children who migrated out of the study area or had incomplete data.

In a third descriptive approach we took, we revealed that about 40% of late adolescent/young adult height was achieved, on average, by mid-infancy, and that by mid-year in their 2^nd^ year of life, children had gained almost half of their young adult height. This display revealed that girls and boys, whose heights were in a normal range (L/HAZ > -1) at six years of age had reached 74% and 69%, respectively, of their young adult height, while children who were stunted at six-years of age (L/HAZ < -2) had achieved less, 68% and 64%, respectively, of their young adult height. Interestingly, this latter finding raises a hypothesis that children who were stunted at six years may have accelerated their school-aged linear growth, relative to children of normal height, to reach their final young adult height. Our findings in children with normal height at six years are similar to a previous report in Denmark where, by age 7 years, girls and boys had reached 73% and 69%, respectively, of their adult height [4]. This comparison suggests that relative attainment of adult height by school entrant age is comparable among normally growing children across different habitats, and that distributions of young adult height may be approximated by age six years.

In the present study, while ranges of age-specific regression coefficients were comparable, more coefficients reflecting young adult height as a function of preschool length or height were larger based on measurements in the same age strata taken sixteen months later. Adjusting for the shorter early childhood to young adult interval length did not affect this observation, suggesting possible unmeasured influences that vary by season or year. The later early childhood measurements were taken in January-April 1991, covering the dry, cool post-rice harvest winter months when vegetables are also more available compared to the timing of the initial measurements, taken from September through December 1989, representing a post-monsoon, pre-harvest, and generally “lean” period of the year (Table 2 and Supplemental Table 2).

Children who had died during the ∼ 16 year interval from end of trial to young adulthood were from poorer families (Table 1). Otherwise, potential biases of excluding children with incomplete data, including young adults known to have been alive but unreachable for measurement, appear to have be minimal based on comparability of baseline factors assessed during the original trial.

## Conclusion

Our findings add evidence to the literature that attained early childhood stature can be informative in predicting young adult height in a rural, chronically undernourished setting in South Asia. Associations revealed were robust to adjustments for other factors that were themselves variably associated with adult height, such as preschool wasting, socioeconomic status and, in girls, timing of menarche and pregnancy history. In such settings, preschooler anthropometric surveys reveal not only current nutritional status but offer predictive information about young adult size.

### Electronic supplementary material

Below is the link to the electronic supplementary material.

Supplementary Material 1

## Data Availability

No datasets were generated or analysed during the current study.

## Footnotes

1 Late adolescent (16–18 years) and young adult (19–23 years) participants are referred to as “young adults” in this paper.

## Abbreviations

L: Recumbent length
Ht: Standing height
HAZ: Height-for-age z-score
LAZ: Length-for-age z-score
WHZ: Weight-for-height z-score
WAZ: Weight-for-age z-score
