# Supplementary material for: Early childhood height is a determinant of young adult stature in rural Nepal
Source: BMC Public Health. 2024 Jul 30;24:2046. doi: 10.1186/s12889-024-19469-8 (PMC11289932; doi:10.1186/s12889-024-19469-8)
Supplement: Supplementary file 1 — Supplementary Material 1 [file 12889_2024_19469_MOESM1_ESM.docx]

**Supplemental Table 1. Characteristics of Study Children at Baseline and at 16-23 Years of Age (Late Adolescence/Young Adulthood) by Year of Preschool Age in a Population Cohort in the Terai of Nepal**

1a) Female

|  | **Age in Years at Enrollment into Original Vitamin A Trial, September-December 1989 (Baseline)** | | | | | | | | | |
| --- | --- | --- | --- | --- | --- | --- | --- | --- | --- | --- |
|  | **< 1** | | **1** | | **2** | | **3** | | **4** | |
|  | **N** | **Mean (SD)**  **or %** | **N** | **Mean (SD)**  **or %** | **N** | **Mean (SD)**  **or %** | **N** | **Mean (SD)**  **or %** | **N** | **Mean (SD)**  **or %** |
| **Baseline status^1^** |  |  |  |  |  |  |  |  |  |  |
| Age, mo | 189 | 5.4 (3.2) | 211 | 17.1 (3.6) | 154 | 29.3 (3.6) | 173 | 40.7 (3.7) | 131 | 53.0 (3.4) |
| L/Ht, cm | 189 | 60.3 (5.9) | 211 | 72.3 (4.7) | 154 | 80.2 (4.4) | 173 | 86.6 (6.0) | 131 | 94.1 (5.6) |
| L/HAZ, per 1 Z | 189 | -1.3 (1.1) | 210 | -2.5 (1.2) | 154 | -2.5 (1.2) | 172 | -2.7 (1.5) | 131 | -2.5 (1.3) |
| WHZ, per 1 Z | 181 | -0.6 (0.9) | 210 | -1.3 (0.9) | 154 | -1.1 (0.7) | 172 | -0.8 (0.8) | 131 | -0.8 (0.8) |
| WAZ, per 1 Z | 189 | -1.4 (1.0) | 210 | -2.4 (1.0) | 154 | -2.4 (0.9) | 172 | -2.2 (1.0) | 131 | -2.1 (0.9) |
| **Status at 16-23 Years of Age^1^** |  |  |  |  |  |  |  |  |  |  |
| Age, mo | 189 | 215.1 (6.1) | 211 | 227.0 (6.0) | 154 | 238.7 (6.1) | 173 | 251.3 (6.1) | 131 | 263.7 (6.1) |
| Age, yr | 189 | 18.0 (0.5) | 211 | 19.0 (0.5) | 154 | 20.0 (0.5) | 173 | 21.0 (0.5) | 131 | 22.0 (0.5) |
| Height, cm | 189 | 151.3 (5.7) | 211 | 150.9 (6.3) | 154 | 152.0 (6.0) | 173 | 150.7 (6.1) | 131 | 151.5 (5.7) |
| Weight, kg | 189 | 45.5 (6.1) | 211 | 45.3 (6.0) | 154 | 45.9 (5.6) | 173 | 45.5 (6.3) | 131 | 46.5 (6.7) |
| BMI, kg/m² | 189 | 19.9 (2.3) | 211 | 19.9 (2.3) | 154 | 19.9 (2.1) | 173 | 20.0 (2.3) | 131 | 20.2 (2.5) |
| **Marriage status** |  |  |  |  |  |  |  |  |  |  |
| Unmarried | 159 | 84.6% | 150 | 72.1% | 100 | 64.9% | 82 | 48.8% | 54 | 42.5% |
| Married^2^ | 28 | 14.9% | 56 | 26.9% | 54 | 35.1% | 84 | 50.0% | 73 | 57.5% |
| Other^3^ | 1 | 0.5% | 2 | 1.0% | 0 | 0% | 2 | 1.2% | 0 | 0% |
| **Ever pregnant among married** | 15 | 53.6% | 30 | 53.6% | 42 | 77.8% | 77 | 91.7% | 68 | 93.2% |
| **Age at menarche, mo^4^** | 178 | 174.9 (13.5) | 200 | 178.1 (16.3) | 151 | 177.4 (13.7) | 161 | 176.6 (16.1) | 122 | 180.2 (15.7) |
| **Time since menarche, mo^4^** | 178 | 40.4 (14.7) | 200 | 48.7 (17.6) | 151 | 61.4 (15.5) | 161 | 74.6 (17.4) | 122 | 83.3 (16.3) |
| **Interval duration, mo^5^** | 189 | 209.6 (5.0) | 211 | 209.7 (4.9) | 154 | 209.4 (5.3) | 173 | 210.6 (5.2) | 131 | 210.6 (5.3) |
| **Caste** |  |  |  |  |  |  |  |  |  |  |
| Brahmin | 29 | 15.3% | 26 | 12.3% | 27 | 17.6% | 21 | 12.1% | 18 | 13.7% |
| Chhetri | 18 | 9.5% | 17 | 8.1% | 18 | 11.7% | 20 | 11.6% | 19 | 14.5% |
| Vaiysha | 116 | 61.4% | 139 | 65.9% | 93 | 60.4% | 109 | 63.0% | 82 | 62.6% |
| Shudra (Dalit) | 20 | 10.6% | 16 | 7.6% | 11 | 7.1% | 13 | 7.5% | 8 | 6.1% |
| Non-Hindu | 6 | 3.2% | 13 | 6.2% | 5 | 3.3% | 9 | 5.2% | 4 | 3.1% |

^1^LAZ, length-for-age z-score; HAZ, height-for-age z-score; WHZ, weight-for-height z-score; WAZ, weight-for-age z-score; BMI, body mass index

^2^Married and living with spouse

^3^Including "Divorced, separated", "Married but not living with spouse" and "don't know"

^4^Girls who haven't menarche yet (N=7) were excluded

^5^Time interval between baseline and late adolescence/young adult assessment

1b) Male

|  | **Age in Years at Enrollment into Original Vitamin A Trial, September-December 1989 (Baseline)** | | | | | | | | | |
| --- | --- | --- | --- | --- | --- | --- | --- | --- | --- | --- |
|  | **< 1** | | **1** | | **2** | | **3** | | **4** | |
|  | **N** | **Mean (SD)**  **or %** | **N** | **Mean (SD)**  **or %** | **N** | **Mean (SD)**  **or %** | **N** | **Mean (SD)**  **or %** | **N** | **Mean (SD)**  **or %** |
| **Baseline status^1^** |  |  |  |  |  |  |  |  |  |  |
| Age, mo | 237 | 5.7 (3.4) | 235 | 17.2 (3.4) | 256 | 29.0 (3.7) | 246 | 41.1 (3.7) | 242 | 53.0 (3.5) |
| L/Ht, cm | 237 | 62.0 (6.6) | 235 | 74.1 (4.3) | 256 | 81.3 (4.4) | 246 | 89.4 (5.8) | 242 | 95.8 (5.2) |
| L/HAZ, per 1 Z | 237 | -1.4 (1.2) | 235 | -2.5 (1.1) | 256 | -2.4 (1.1) | 245 | -2.3 (1.3) | 242 | -2.3 (1.1) |
| WHZ, per 1 Z | 230 | -0.5 (0.9) | 235 | -1.5 (0.9) | 256 | -1.0 (0.8) | 245 | -0.9 (0.8) | 242 | -0.9 (0.7) |
| WAZ, per 1 Z | 237 | -1.4 (1.2) | 235 | -2.5 (0.9) | 256 | -2.2 (0.9) | 245 | -2.0 (0.9) | 242 | -2.0 (0.7) |
| **Status at 16-23 Years of Age^1^** |  |  |  |  |  |  |  |  |  |  |
| Age, mo | 237 | 216.3 (6.4) | 235 | 227.8 (6.6) | 256 | 239.8 (6.5) | 246 | 253.4 (6.5) | 242 | 265.2 (6.2) |
| Age, yr | 237 | 18.1 (0.5) | 235 | 19.0 (0.5) | 256 | 20.0 (0.5) | 246 | 21.2 (0.5) | 242 | 22.2 (0.5) |
| Height, cm | 237 | 161.9 (6.3) | 235 | 163.1 (6.3) | 256 | 163.5 (6.2) | 246 | 163.3 (6.9) | 242 | 164.2 (6.4) |
| Weight, kg | 237 | 49.9 (6.3) | 235 | 51.2 (6.5) | 256 | 51.8 (6.1) | 246 | 52.4 (7.1) | 242 | 52.8 (7.1) |
| BMI, kg/m² | 237 | 19.0 (1.8) | 235 | 19.3 (2.1) | 256 | 19.4 (2.0) | 246 | 19.6 (2.1) | 242 | 19.5 (2.2) |
| **Marriage status** |  |  |  |  |  |  |  |  |  |  |
| Unmarried | 208 | 91.2% | 199 | 88.4% | 189 | 77.5% | 149 | 63.4% | 132 | 57.4% |
| Married^2^ | 20 | 8.8% | 25 | 11.1% | 55 | 22.5% | 84 | 35.7% | 97 | 42.2% |
| Other^3^ | 0 | 0% | 1 | 0.4% | 0 | 0% | 2 | 0.9% | 1 | 0.4% |
| **Ever pregnant among married** | - | - | - | - | - | - | - | - | - | - |
| **Age at menarche, mo^4^** | - | - | - | - | - | - | - | - | - | - |
| **Time since menarche, mo^4^** | - | - | - | - | - | - | - | - | - | - |
| **Interval duration, mo^5^** | 237 | 210.4 (5.2) | 235 | 210.5 (5.2) | 256 | 210.8 (5.2) | 246 | 212.1 (5.2) | 242 | 212.0 (5.2) |
| **Caste** |  |  |  |  |  |  |  |  |  |  |
| Brahmin | 26 | 11.0% | 34 | 14.5% | 31 | 12.1% | 27 | 11.0% | 32 | 13.2% |
| Chhetri | 21 | 8.9% | 23 | 9.8% | 24 | 9.4% | 22 | 8.9% | 21 | 8.7% |
| Vaiysha | 165 | 69.6% | 156 | 66.4% | 168 | 65.6% | 166 | 67.5% | 147 | 60.7% |
| Shudra (Dalit) | 12 | 5.1% | 12 | 5.1% | 29 | 7.8% | 18 | 7.3% | 27 | 11.2% |
| Non-Hindu | 12 | 5.1% | 9 | 3.8% | 13 | 5.1% | 13 | 5.3% | 15 | 6.2% |

^1^LAZ, length-for-age z-score; HAZ, height-for-age z-score; WHZ, weight-for-height z-score; WAZ, weight-for-age z-score; BMI, body mass index.

^2^Married and living with spouse.

^3^Including "Divorced, separated" and "Married but not living with spouse"

^4^Time interval between baseline and young adult assessment

**Notes for Supplemental Table 1.**

For girls and boys, low mean L/HAZ was evident in infancy (~ -1.35), at age 1 (-2.5), 2 (~ -2.45), 3 (-2.7 and -2.3, respectively), and 4 (-2.5 and -2.3, respectively). WHZ was mild-to-moderately low in both sexes, with means of ~ -0.55, -1.45, -1.05, -0.85, and -0.85. Across the five age groups, young women plateaued in height (mean of ~151 cm), weighing a mean of ~45.7 kg with a mean BMI of ~20. Young men continued to increase in height (from 161.9 to 164.2 cm) and weight (means of 49.9 to 52.8 kg), yielding a mean BMI of 19 to 19.6. Young women reported menarche to have occurred at ~177 months (14.8 yr). The percentage married increased sharply with age, from 14.9% to 57.5%, among whom over half to over 90% reported a history of ever pregnant. Nearly 2/3s of all households of participants belonged to the Vaiysha caste, comprising agricultural workers and local merchants.

**Supplemental Figure 1. Flowchart of participants as preschoolers at 16-month visit (in 1991), and as censused (in 2006) and followed-up as late adolescents and young adults (in 2006-2008), Nepal Nutrition Intervention Project-Sarlahi, Nepal.**

**Supplemental Table 2. Early Childhood Length or Height^1^ (L/Ht, cm) at 16-month Original Vitamin A Trial Visit and its Association by Age and Sex with Height (cm) at 16-23 Years of age in a Population Cohort in the Terai of Nepal**

| **Preschool age (yr)** | **N** | | **Height at 16-month visit**  **Mean (SD)** | | **Young adult height Mean (SD)** | | **Young adult age**  **in month**  **Median (IQR)** | | **Young adult height/preschool L/Ht in cm**  **[b (SE)]** | | | |
| --- | --- | --- | --- | --- | --- | --- | --- | --- | --- | --- | --- | --- |
|  | **Female** | **Male** | **Female** | **Male** | **Female** | **Male** | **Female** | **Male** | **Female** | | **Male** | |
|  |  |  |  |  |  |  |  |  | **Model 1^2^** | **Model 2^3^** | **Model 1^2^** | **Model 2^3^** |
| 1 | 116 | 143 | 74.2 (3.3) | 75.8 (3.6) | 151.1 (5.5) | 161.7 (6.7) | 213 (208-218) | 214 (209-219) | 1.01 (0.13) * | 1.06 (0.16) * | 1.13 (0.14) * | 1.06 (0.15) * |
| 2 | 200 | 239 | 80.1 (4.1) | 81.3 (4.6) | 150.9 (5.7) | 163.0 (6.2) | 223 (219-227) | 223 (219-229) | 1.04 (0.08) * | 1.00 (0.09) * | 1.13 (0.07) * | 1.09 (0.07) * |
| 3 | 172 | 231 | 88.1 (4.5) | 89.0 (4.2) | 152.1 (6.8) | 163.1 (5.9) | 235 (230-239) | 236 (232-240) | 1.15 (0.09) * | 1.16 (0.09) * | 0.86 (0.08) * | 0.89 (0.08) * |
| 4 | 170 | 228 | 94.0 (5.6) | 95.3 (5.4) | 151.3 (5.5) | 163.4 (6.4) | 247 (242-252) | 249 (244-254) | 0.53 (0.07) * | 0.49 (0.07) * | 0.76 (0.07) * | 0.74 (0.07) * |
| 5 | 130 | 228 | 99.7 (6.5) | 102.7 (5.5) | 150.7 (8.4) | 164.1 (6.7) | 260 (255-263) | 262 (257-266) | 0.42 (0.11) * | 0.50 (0.12) * | 0.69 (0.07) * | 0.66 (0.07) * |
| 6 | 48 | 94 | 103.3 (5.6) | 105.7 (5.1) | 149. 6 (4.4) | 163.4 (7.0) | 265 (263-271) | 271 (265-273) | 0.50 (0.10) * | 0.46 (0.11) * | 0.73 (0.12) * | 0.61 (0.12) * |

^1^Recumbent length (L) for children aged <24 months. Standing height (Ht) for children > 24 months.

^2^Coefficient adjusted for interval duration in months and older adolescent/young adult age in months

^3^Coefficient further adjusted for caste, preschool weight-for-height (WHZ) z-score, and for female, also included time since menarche in month, and marriage and pregnancy status (see supplemental table 1 for distributions)

*p<0.001, †p<0.01, ‡p<0.05

**Supplemental Table 3. Association of Length or Height for Age Z-score (L/HAZ) at 16-month Original Vitamin A Trial Follow-up Visit with Height (cm) at 16-23 Years of Age in a Population Cohort in the Terai of Nepal**

| **Model Covariates** | **Girl** | | | | **Boy** | | | |
| --- | --- | --- | --- | --- | --- | --- | --- | --- |
|  | **Model 1^1^** | | **Model 2^2^** | | **Model 1^1^** | | **Model 2^2^** | |
|  | **N=836** | | **N=783** | | **N=1163** | | **N=1163** | |
|  | **b (SE)** | **P-value** | **b (SE)** | **P-value** | **b (SE)** | **P-value** | **b (SE)** | **P-value** |
| **L/HAZ** | 3.00 (0.16) | <0.001 | 2.95 (0.16) | <0.001 | 3.51 (0.14) | <0.001 | 3.39 (0.14) | <0.001 |
| **Young adult age, mo** | -0.02 (0.01) | 0.10 | 0.02 (0.02) | 0.23 | 0.01 (0.01) | 0.13 | 0.02 (0.01) | 0.03 |
| **Interval duration, mo^3^** | 0.03 (0.04) | 0.48 | 0.03 (0.04) | 0.42 | -0.0003 (0.03) | 0.99 | 0.005 (0.03) | 0.86 |
| **Marriage and pregnancy status^4^** |  |  |  |  |  |  |  |  |
| married but not pregnant | - | - | 0.66 (0.76) | 0.38 | - | - | - | - |
| married and pregnant | - | - | -0.37 (0.47) | 0.44 | - | - | - | - |
| **Time since menarche, mo^5^** | - | - | -0.04 (0.01) | 0.001 | - | - | - | - |
| **Preschool WHZ** | - | - | 0.13 (0.24) | 0.58 | - | - | -0.23 (0.20) | 0.25 |
| **Caste^6^** |  |  |  |  |  |  |  |  |
| Chhetri | - | - | -0.16 (0.74) | 0.83 | - | - | -0.64 (0.66) | 0.34 |
| Vaiysha | - | - | -1.93 (0.56) | 0.001 | - | - | -2.21 (0.47) | <0.001 |
| Shudra | - | - | -4.41 (0.86) | <0.001 | - | - | -5.42 (0.70) | <0.001 |
| Non-Hindu | - | - | -2.77 (1.10) | 0.01 | - | - | -2.25 (0.78) | 0.004 |
| **cons_** | 157.72 (7.1) | <0.001 | 152.07 (7.6) | <0.001 | 168.41 (5.6) | <0.001 | 167.57 (5.6) | <0.001 |

^1^Coefficient adjusted for interval duration in months and older adolescent/young adult age in months

^2^Coefficient further adjusted for caste, preschool weight-for-height (WHZ) z-score, and for female, also included time since menarche in month, and marriage and pregnancy status (see supplemental table 1 for distributions)

^3^Time interval between 16-month follow-up and young adult assessment

^4^“Neither married nor pregnant” was the comparison group. Adjusted mean young adult heights by marriage and pregnancy status were listed in Supplemental Table 5.

^5^Girls who haven't menarche yet (N=7) were excluded in Model 2

^6^“Brahmin” was the comparison group. Adjusted mean young adult heights by caste were listed in Supplemental Table 5.

**Supplemental Figure 2. Percent of Height at 16-23 Years of Age Attained by Year of Early Childhood Age at 16-month Visit, by Sex and Preschool Length/Height for Age Z-score in a Population Cohort in the Terai of Nepal**

1. **Female**

**
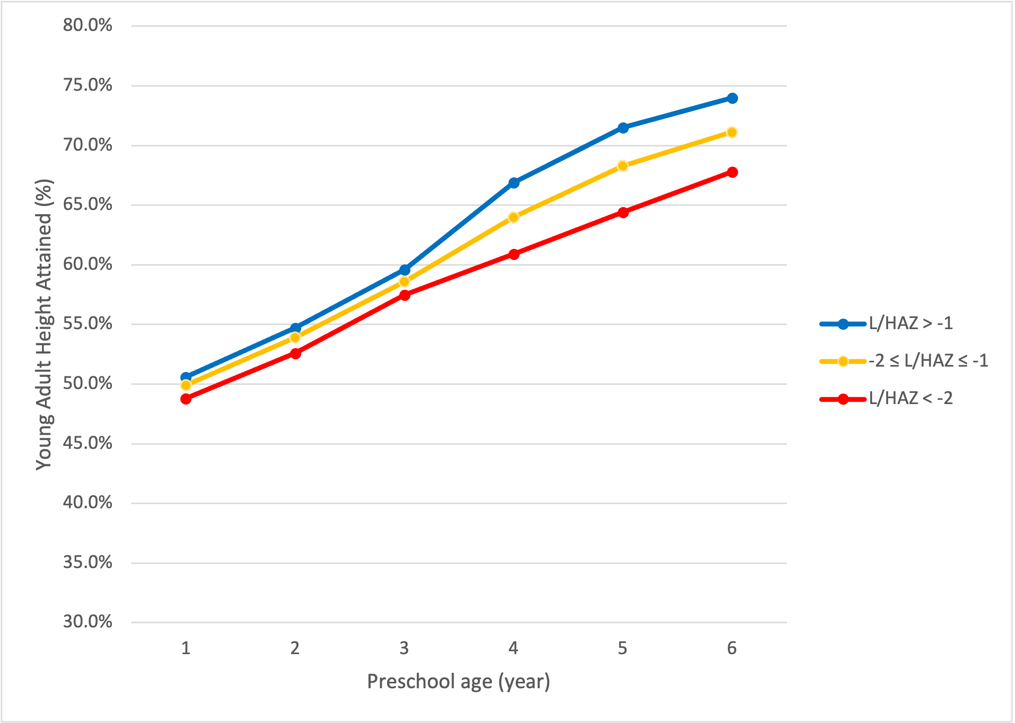
**

1. **Male**

**
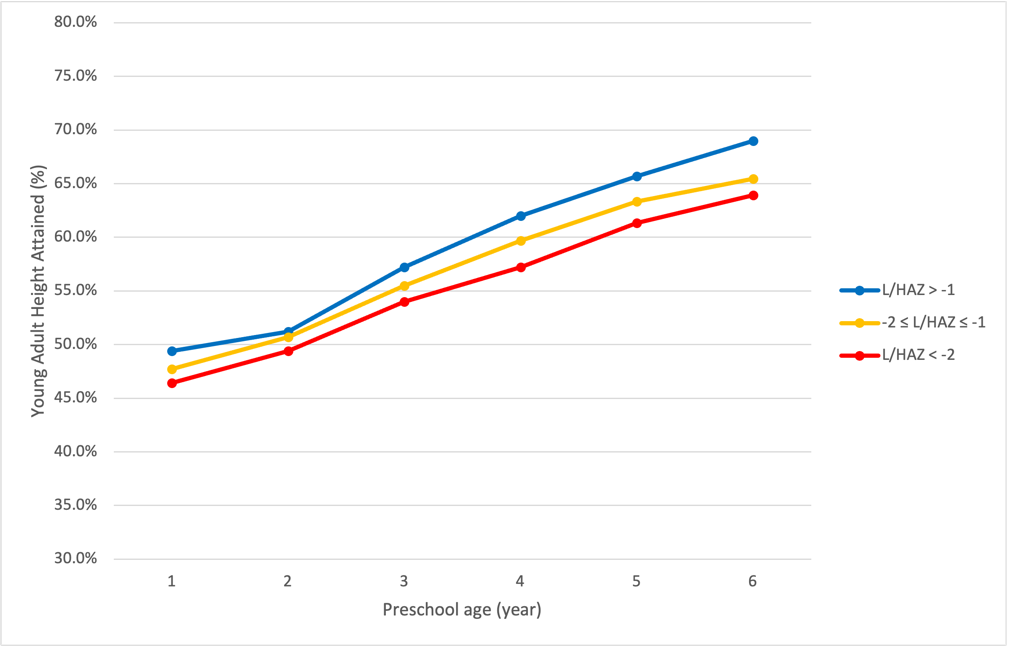
**

**Supplemental Table 4. Young Adult Height by Caste, Marital Status, and Pregnancy History at Baseline and 16-month Visit in the Original Trial by Sex.**

|  | **Adjusted Young Adult Height, mean (SE)** | | | |
| --- | --- | --- | --- | --- |
|  | **Model starting with baseline assessment^1^** | | **Model starting with 16-month visit assessment^2^** | |
|  | **Female** | **Male** | **Female** | **Male** |
|  | **N=798** | **N=1206** | **N=783** | **N=1163** |
| **Caste** |  |  |  |  |
| Brahmin | 153.52 (0.49) | 165.56 (0.45) | 152.84 (0.51) | 165.25 (0.43) |
| Chhetri | 152.70 (0.56) | 164.89 (0.52) | 152.68 (0.56) | 164.62 (0.51) |
| Vaiysha | 150.98 (0.23) | 162.98 (0.20) | 150.92 (0.23) | 163.04 (0.18) |
| Shudra | 148.07 (0.67) | 160.15 (0.59) | 148.43 (0.66) | 159.83 (0.56) |
| Non-Hindu | 149.07 (0.90) | 162.23 (0.70) | 150.07 (0.95) | 163.00 (0.65) |
| **Marriage and pregnancy status** |  |  |  |  |
| neither married nor pregnant | 151.27 (0.24) | - | 151.20 (0.24) | - |
| married but not pregnant | 152.07 (0.70) | - | 151.86 (0.71) | - |
| married and pregnant | 150.93 (0.38) | - | 150.83 (0.38) | - |

^1^Estimates derived from Model 2 in Table 3.

^2^Estimates derived from Model 2 in Supplemental Table 3.

**Supplemental Table 5. Percentage of Attained Height at 16-23 Years of Age at Baseline and 16-month Visit in the Original Trial by Preschool Age, Sex and Preschool Length or Height for Age Z-score (L/HAZ) in a Population Cohort in the Terai of Nepal**

1. **Baseline**

| **Preschool age (yr)** | **Female** | | | | | | **Male** | | | | | |
| --- | --- | --- | --- | --- | --- | --- | --- | --- | --- | --- | --- | --- |
|  | **L/HAZ < -2** | | **-2 ≤ L/HAZ ≤ -1** | | **L/HAZ > -1** | | **L/HAZ < -2** | | **-2 ≤ L/HAZ ≤ -1** | | **L/HAZ > -1** | |
|  | **N** | **Attained Height** | **N** | **Attained Height** | **N** | **Attained Height** | **N** | **Attained Height** | **N** | **Attained Height** | **N** | **Attained Height** |
| **<1** | 51 | 40.2% | 65 | 40.6% | 73 | 39.1% | 78 | 38.4% | 71 | 38.9% | 88 | 37.8% |
| **1** | 146 | 47.5% | 41 | 48.5% | 24 | 49.7% | 163 | 45.0% | 49 | 45.9% | 23 | 47.4% |
| **2** | 105 | 52.4% | 31 | 53.1% | 18 | 55.1% | 161 | 49.0% | 69 | 50.5% | 26 | 52.6% |
| **3** | 119 | 55.8% | 32 | 59.9% | 22 | 63.4% | 143 | 53.4% | 66 | 55.6% | 37 | 58.5% |
| **4** | 82 | 60.3% | 32 | 64.4% | 17 | 66.7% | 155 | 57.3% | 57 | 59.3% | 30 | 62.4% |

1. **16-month visit**

| **Preschool age (yr)** | **Female** | | | | | | **Male** | | | | | |
| --- | --- | --- | --- | --- | --- | --- | --- | --- | --- | --- | --- | --- |
|  | **L/HAZ < -2** | | **-2 ≤ L/HAZ ≤ -1** | | **L/HAZ > -1** | | **L/HAZ < -2** | | **-2 ≤ L/HAZ ≤ -1** | | **L/HAZ > -1** | |
|  | **N** | **Attained Height** | **N** | **Attained Height** | **N** | **Attained Height** | **N** | **Attained Height** | **N** | **Attained Height** | **N** | **Attained Height** |
| **1** | 87 | 48.8% | 24 | 49.9% | 5 | 50.6% | 104 | 46.4% | 29 | 47.7% | 10 | 49.4% |
| **2** | 142 | 52.6% | 45 | 53.9% | 13 | 54.7% | 163 | 49.4% | 56 | 50.7% | 20 | 51.2% |
| **3** | 114 | 57.5% | 41 | 58.6% | 17 | 59.6% | 153 | 54.0% | 63 | 55.5% | 15 | 57.2% |
| **4** | 120 | 60.9% | 31 | 64.0% | 19 | 66.9% | 141 | 57.2% | 67 | 59.7% | 20 | 62.0% |
| **5** | 80 | 64.4% | 33 | 68.3% | 17 | 71.5% | 126 | 61.3% | 64 | 63.3% | 38 | 65.7% |
| **6** | 32 | 67.8% | 13 | 71.1% | 3 | 74.0% | 59 | 63.9% | 28 | 65.5% | 7 | 69.0% |
